# Gastroenteropancreatic neuroendocrine neoplasms in children and adults: a comparative review

**DOI:** 10.3389/fendo.2025.1736543

**Published:** 2026-01-21

**Authors:** Michaela Kuhlen, Rainer Claus, Marianne E. Pavel, Marina Kunstreich, Nehara Begum, Constantin Lapa, Antje Redlich

**Affiliations:** 1 Pediatrics and Adolescent Medicine, Faculty of Medicine, University of Augsburg, Augsburg, Germany; 2 Bavarian Cancer Research Center (BZKF), Augsburg, Germany; 3 Pathology, Faculty of Medicine, University of Augsburg, Augsburg, Germany; 4 Department of Medicine 1, Uniklinikum Erlangen and Comprehensive Cancer Center CCC-EMN, Friedrich Alexander University Erlangen-Nürnberg, Erlangen, Germany; 5 Department of Pediatrics, Pediatric Hematology/Oncology, Otto-von-Guericke-University, Magdeburg, Germany; 6 Department for General-, Visceral-, Thoracic- and Endocrine Surgery, European Neuroendocrine Tumor Society (ENETS)-Center of Excellence, Johannes-Wesling-Klinikum Minden, University Hospital of the Ruhr-University Bochum, Minden, Germany; 7 Nuclear Medicine, Faculty of Medicine, University of Augsburg, Augsburg, Germany

**Keywords:** adults, children and adolescents, gastroenteropancreatic, management, neuroendocrine neoplasms

## Abstract

**Background:**

Gastroenteropancreatic neuroendocrine neoplasms (GEPNENs) represent a biologically heterogeneous tumor group that is increasingly recognized in adults but remains exceptionally rare in children. While adult management is guided by evidence-based recommendations, pediatric practice relies mainly on registry data and extrapolation. A direct comparison is needed to identify shared principles, highlight divergences, and define research priorities.

**Methods:**

We performed a structured literature review of pediatric GEPNENs (pancreatic, gastrointestinal [excluding appendix], and neuroendocrine neoplasms of unknown primary) and contrasted these findings with adult guidelines (ENETS 2023–2024, ESMO 2020–2024, ASCO 2023, NANETS 2018-2023) and pivotal clinical trials. Domains analyzed included epidemiology, clinical presentation, histological and molecular characteristics, treatment strategies, outcomes, and guideline frameworks.

**Results:**

Pediatric GEPNENs are strongly enriched for hereditary cancer predisposition syndromes (MEN1, VHL, NF1, TSC) and show a predominance of well-differentiated NET G1–G2. In contrast, adults exhibit the full spectrum of NET G1–3 and NEC G3. Somatostatin receptor (SSTR) expression is frequent in both pediatric and adult NETs, supporting the use of somatostatin analogues (SSAs) and peptide receptor radionuclide therapy (PRRT) in advanced disease; SSTR expression declines with increasing grade. Surgical resection remains the only curative option in both populations, with pediatric practice prioritizing organ preservation and minimization of late effects. In adults, systemic therapy sequencing is structured by randomized trials, whereas pediatric use of systemic therapies is adapted case-by-case, with emerging but still limited evidence. Survival in localized pediatric NETs exceeds 90%, but remains poor in metastatic and high-grade disease, similar to adults.

**Conclusions:**

Although histological frameworks are shared, pediatric GEPNENs differ from adult disease in genetics, site distribution, functional status, and survivorship challenges. Adult evidence may be cautiously adapted to pediatrics, but pediatric-specific guidelines and collaborative research are urgently needed to address unique biological and clinical features and to harmonize long-term care.

## 1 Introduction

Gastroenteropancreatic neuroendocrine neoplasms (GEPNENs) comprise a biologically and clinically heterogeneous spectrum of tumors arising from neuroendocrine cells in the gastrointestinal tract, pancreas, and—very rarely—bile duct and gall bladder (1). According to the World Health Organization (WHO) classification (5th Edition, 2019), GEPNENs are stratified into well-differentiated neuroendocrine tumors (NETs, grades 1–3) and poorly differentiated neuroendocrine carcinomas (NECs, grade 3), recognizing NET G3 as a distinct entity with implications for prognosis and management (2–4).

In adults, GEPNEN care is now structured by detailed consensus guidance. ENETS (5–8) has published organ-specific recommendations for pancreatic, small intestinal, gastric, and colorectal NETs, as well as digestive NECs, alongside complementary ESMO (9) publications, NANETS (10, 11) guidelines on unresectable, metastatic, or high-grade GEPNEN, and ASCO’s (12) guideline on systemic therapy for metastatic NETs. These frameworks define diagnostic standards, staging algorithms, and therapeutic sequencing—including surgery, somatostatin analogues (SSAs), peptide receptor radionuclide therapy (PRRT), targeted drugs, chemotherapy, and liver-directed interventions.

By contrast, pediatric GEPNENs are extremely rare. Current evidence derives mainly from national and European registries [German MET (13–15), Italian TREP (16, 17), French FRACTURE (18)] and population-based cohorts [Surveillance, Epidemiology, and End Results (SEER) (19, 20)]. These series consistently demonstrate that NET G1–G2 predominate in children, while NET G3 and NEC G3 are uncommon. Curative management relies on surgical resection; systemic therapies (SSA, PRRT, everolimus, sunitinib, chemotherapy) are used in advanced cases but are largely adapted from adult protocols, with only emerging pediatric experience (21). Evidence for high-grade disease and liver-directed approaches remains particularly sparse.

Epidemiological patterns also diverge between adults and children. In adults, incidence of GEPNENs has increased steadily over recent decades—largely due to greater detection and stage migration—resulting in a growing prevalent population and changing therapeutic needs (22). In pediatrics, incidence remains stable and extremely low (23). Moreover, a comparatively high proportion of pediatric GEPNENs are associated with hereditary cancer predisposition syndromes, underscoring the distinct biology of childhood disease (13, 14, 24).

Appendiceal and bronchopulmonary NENs represent the most frequent pediatric subtypes with unique clinical features, but both are already covered by dedicated ESCP (European Standard Clinical Practice) guidance documents (25). Therefore, they are not included in this review. Instead, we focus on non-appendiceal, non-bronchopulmonary GEPNENs, namely pancreatic NENs, gastrointestinal NENs (gastric, duodenal, small intestinal, colorectal, Meckel’s), and pediatric NEN-CUP (NEN of unknown primary).

### 1.1 Rationale for a pediatric–adult comparison

Despite common histopathological categories, important biological and clinical differences exist between children and adults with GEPNENs. These include differences in genetic predisposition, distribution of primary sites, functional status at presentation, patterns of metastatic spread, and long-term outcomes. In addition, while adult treatment algorithms are supported by randomized or prospective trials, pediatric treatment remains based on small series and extrapolation.

**Figure 1:**
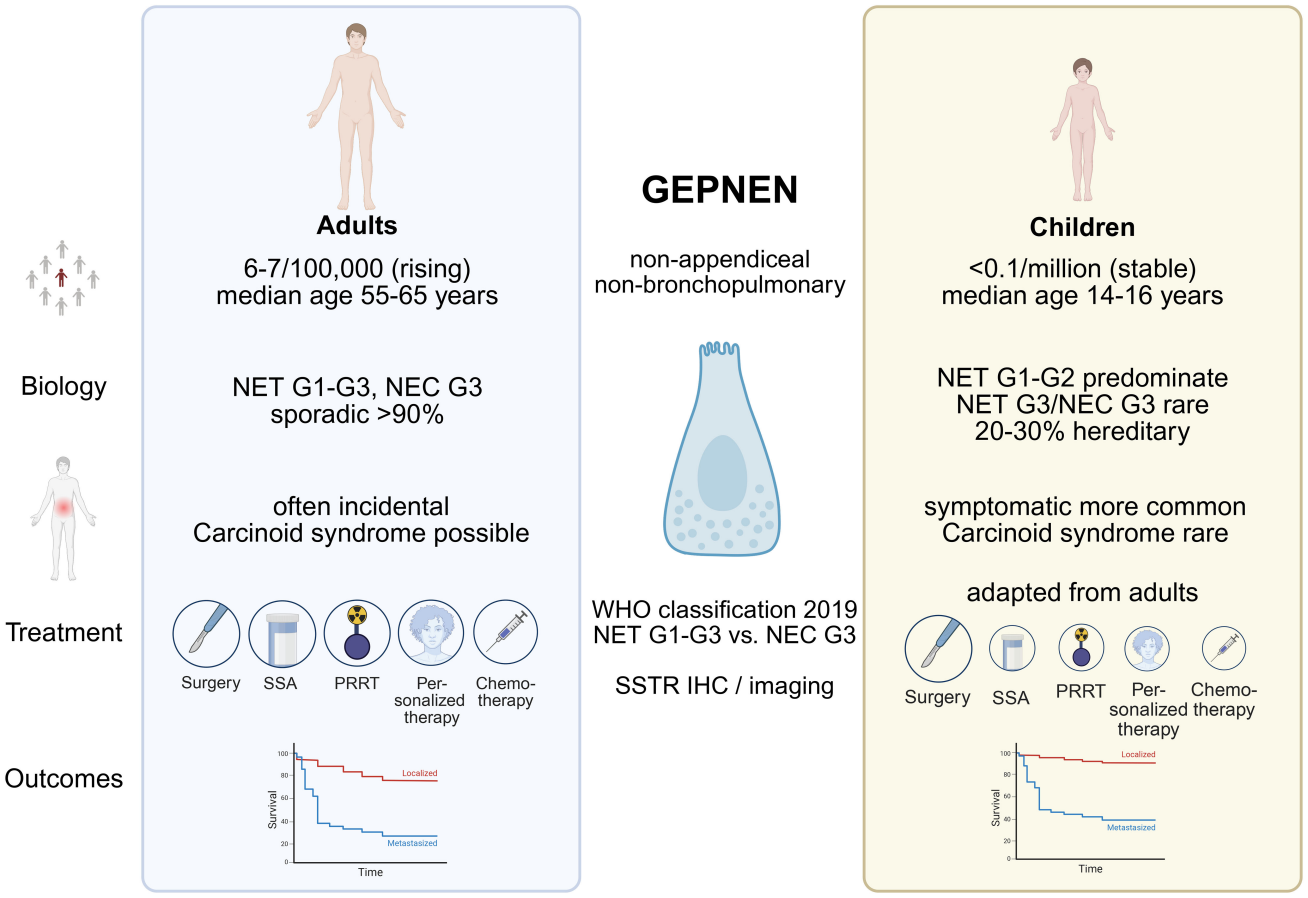
Pediatric and adult GEPNENs: A comparative visual guide Created in https://BioRender.com.

A structured comparative synthesis is therefore needed to: (i) delineate where pediatric biology and (**Figure 1**) clinical course diverge from adult disease; (ii) identify which elements of adult guideline recommendations can be safely translated to pediatrics; and (iii) highlight areas where pediatric-specific evidence is lacking and future collaborative studies are most urgently required.

## 2 Methods

This comparative review was conducted to synthesize the available evidence on pediatric GEPNENs and to contrast these findings with established adult data and guideline-based recommendations.

### 2.1 Literature search and sources

A structured literature review was performed in PubMed/MEDLINE, Embase, and Web of Science for publications up to August 2025. Search terms included combinations of “neuroendocrine neoplasm”, “neuroendocrine tumor”, “pancreatic NET”, “gastrointestinal NET”, “hepatic NET”, “unknown primary”, “pediatric”, “adolescent”, and “child”. Reference lists of key studies and guidelines were screened manually to identify additional relevant reports.

### 2.2 Inclusion and exclusion criteria

Inclusion: Studies reporting original data on children and adolescents (<18 years) with histologically confirmed non-appendiceal GEPNENs, including pancreatic, gastric, duodenal, small-intestinal, colorectal, Meckel’s diverticulum, and NEN-CUP. Both registry-based and institutional series were eligible.

Exclusion: Appendiceal and bronchopulmonary NENs were excluded. Case reports were not systematically reviewed but used selectively to illustrate rare clinical scenarios.

For adult comparators, evidence was drawn primarily from current international guidelines: ENETS 2023–2024 site-specific recommendations for pancreatic, gastroduodenal, small intestinal, and colorectal NETs, and digestive NECs (5–8, 26). ESMO Clinical Practice Guidelines (latest available updates, 2020–2024) (9, 27). NANETS (2018–2023) guidelines on unresectable, metastatic, or high-grade GEPNEN (10, 11) ASCO 2023 guideline on systemic therapy for metastatic well-differentiated GEP-NETs (12).

Additional large-scale epidemiological analyses (e.g., SEER, European registries) and pivotal clinical trials were also included to contextualize treatment strategies and outcomes.

### 2.3 Data synthesis and comparative framework

Given the rarity of pediatric GEPNENs and the absence of prospective trials, evidence synthesis relied heavily on: Registry data from the German Malignant Endocrine Tumor (MET) Registry (13, 14, 15, 28), the Italian TREP Project (16, 17), the French FRACTURE Registry (18), and North American SEER analyses (19, 20). retrospective series addressing pediatric pancreatic NENs, gastrointestinal NENs, and NEN-CUP (29–31), and narrative and systematic reviews, including those evaluating systemic therapy in pediatric NENs (21, 32–34).

Adult and pediatric data were then compared across the following domains: Epidemiology and clinical presentation, histological and molecular characteristics, treatment strategies (surgery, systemic therapy, liver-directed interventions, other approaches), and prognosis and long-term outcomes, and guideline recommendations and current clinical practice.

This framework was chosen to mirror the structure of existing adult guidelines and to facilitate direct comparison, highlighting both overlaps and divergences in disease biology, therapeutic approaches, and outcomes.

## 3 Epidemiology and clinical presentation differences

### 3.1 Incidence and demographics

In adults, the incidence of GEPNENs has risen markedly over the past four decades. Population-based data from the SEER program and European registries document a 6- to 7-fold increase since the 1970s, with a current age-adjusted incidence of approximately 6–7 per 100,000 per year (22, 35–38). This rise is largely attributed to increased use of imaging, endoscopy, and histopathological recognition, resulting in a growing prevalence of indolent, localized NENs at diagnosis (38, 39). The median age at presentation in adults is typically in the fifth to sixth decade of life, with no consistent sex predominance across all sites, though pancreatic NENs show a slight male preponderance in some cohorts (37, 38).

In children and adolescents, GEPNENs are exceedingly rare, with an incidence estimated at <0.1 per million per year (23). Registry analyses from the German MET registry, the TREP Project, and FRACTURE confirm that pediatric cases represent <2% of all NENs (13, 14, 16–18, 40–43). The median age at diagnosis is mid-adolescence (14–16 years), with rare cases in younger children, the youngest published case being six years old (13). Unlike adults, where sporadic cases dominate (38), up to 20–30% of pediatric GEPNENs occur in the context of hereditary cancer predisposition syndromes, particularly multiple endocrine neoplasia type 1 (MEN1), von Hippel–Lindau (VHL), neurofibromatosis type 1 (NF1), and tuberous sclerosis complex (TSC) (14, 24).

The distribution of primary sites also differs: in adults, the small intestine and pancreas are the most common non-appendiceal GEPNEN sites (38, 39); in children, pancreatic NENs predominate among non-appendiceal primaries, followed by gastric, duodenal, and colorectal NENs, while primary hepatic NENs are extraordinarily rare (31–34, 44–46). Pediatric NEN -CUP is also reported, but accounts for <5% of all cases (14). A comparative overview on epidemiological features is presented in **Table 1**.

**Table 1:** Comparative epidemiology of GEPNENs in adults vs. children.

Feature	Adults	Children/adolescents
Incidence	~6–7 per 100,000/year (rising steadily)	<0.1 per million/year (stable, extremely rare)
Median age	55–65 years	14–16 years
Sex distribution	Balanced overall; slight male predominance in pancreatic NETs	No consistent sex predominance
Common primary sites (non-appendiceal)	Small intestine, pancreas, rectum	Pancreas > stomach/duodenum > colorectal; small intestine rare
Hereditary syndromes	<10% (mostly MEN1, VHL)	20–30% (MEN1, VHL, NF1, TSC)
Appendiceal NET	Common in adults (esp. incidental)	Most common pediatric NEN, but excluded from this review

NET, neuroendocrine tumor; NEC, neuroendocrine carcinoma; MEN1, multiple endocrine neoplasia type 1, von-Hippel-Lindau Syndrome; NF1, neurofibromatosis type 1; TSC, tuberous sclerosis.

### 3.2 Clinical presentation

Adults most frequently present with non-specific abdominal pain, altered bowel habits, or incidental findings during imaging or endoscopy. Functioning tumors account for 10-30% of NENs, and when present, carcinoid syndrome (flushing, diarrhea, wheezing) is a hallmark of serotonin-producing midgut NENs with hepatic metastases. Functional pancreatic NETs (predominantly insulinoma, gastrinomas, more rarely glucagonomas, VIPomas and others) occur but account for a small proportion of adult cases (37–39).

In children, presentation differs significantly. Functional pancreatic NETs are relatively more frequent than in adults, with insulinomas being the single most common functional subtype (13, 16–18). These present with recurrent hypoglycemia, seizures, or neuroglycopenic symptoms. Gastrinomas are also reported in pediatric MEN1 cohorts, but VIPomas and glucagonomas remain exceptionally rare. Non-functional pancreatic NENs in children often manifest as abdominal pain, palpable mass, or incidental imaging findings (13, 17, 18).

Gastrointestinal NENs in children usually present with abdominal pain, vomiting, melena/hematochezia, or bowel obstruction (15, 17). Carcinoid syndrome is extremely rare in pediatric patients, reflecting the low incidence of serotonin-producing midgut NENs. Liver involvement generally represents metastasis from an occult gastrointestinal primary (14, 32, 33). Pediatric NEN-CUP presents with advanced disease, usually involving the liver, lymph nodes, or bone, and non-specific systemic symptoms (14).

### 3.3 Differences in early detection

The rise in adult GEPNEN incidence is closely linked to increased incidental detection during abdominal imaging or endoscopic evaluation for unrelated indications and surveillance (37–39). Consequently, a growing proportion of adults are diagnosed with small, localized, and indolent tumors before symptoms develop. Older SEER and population-based surveys reported ~50-60% advanced (regional/distant) disease at diagnosis, whereas more recent SEER analyses suggest this proportion has declined to roughly 40-45%, reflecting increased detection of localized cases (35, 47–49).

In children, early or incidental detection is less common. Most pediatric GEPNENs are identified when symptomatic or during work-up for hereditary syndromes (13, 14, 17, 18). Across recent multicenter and registry cohorts, roughly one-third of non-appendiceal pediatric NETs present with distant metastases at diagnosis, with variation by site and biology. Pediatric GEP-NECs—particularly of pancreatic origin—more frequently present with metastatic disease (13, 15, 17, 18).

## 4 Histological and molecular characteristics

### 4.1 Histologic subtypes

The WHO 2019 classification defines GEPNENs based on differentiation (well vs poorly differentiated) and grade (G1–G3, determined by mitotic count and Ki-67 index). Within this framework: Well-differentiated NETs are subdivided into NET G1, G2, and G3, with increasing proliferative activity but preserved neuroendocrine morphology. Poorly differentiated NECs encompass small-cell and large-cell subtypes, which are biologically distinct from NET G3 and generally more aggressive.

In adults, the entire spectrum from NET G1 to NET is encountered, though distribution varies by site. NET G1 and G2 and NEC dominate small intestinal and gastric NETs, while pancreatic NETs more often include NET G2 and G3. NEC is relatively more frequent in adults, accounting for up to 10–15% of GEPNENs and carries a poor prognosis.

In children, available registry data demonstrate a predominance of well-differentiated NET G1 and G2, which account for the vast majority of cases. NET G3 is reported but rare, and NEC is exceptional, with only isolated cases in registry analyses. Importantly, the biological behavior of pediatric NETs often appears more indolent than in adults, even when proliferative indices approach those of NET G2 or low NET G3. This highlights potential differences in tumor biology across age groups.

Site-specific distribution also differs between children and adults. In adults, small intestinal NENs are the most frequent, followed by pancreatic and rectal NENs. In children, pancreatic NENs are the most frequent non-appendiceal subtype, followed by gastric/duodenal NENs. Midgut NENs are relatively rare in pediatrics, and carcinoid syndrome is exceptional. Primary hepatic NENs are extraordinarily rare in both groups, though in adults they have been described slightly more often; in children, nearly all hepatic NENs represent metastases from another primary.

### 4.2 Somatostatin receptor expression

Somatostatin receptor (SSTR) expression represents a clinically relevant biomarker across age groups. In adults, strong membranous expression of SSTR2 (and to a lesser extent SSTR5) is characteristic of well-differentiated NETs, underpinning the efficacy of both SSA and PRRT. Expression declines with increasing grade, and NECs mostly lack sufficient SSTR density for targeted imaging or therapy. In children, systematic data are limited, but registry cohorts and case series confirm a similarly high prevalence of SSTR2 expression in NET G1–2, enabling accurate detection with 68Ga-DOTA–labelled PET imaging and supporting the feasibility of SSA and PRRT in pediatric practice. As in adults, strong and homogenous expression is less common in NET G3 and is rarely present in NEC G3, thereby influencing treatment decisions. Nevertheless, the NETTER-2 trial demonstrated that a subset of well-differentiated NET G3—including adolescents aged 15 years and older—can exhibit strong and homogenous SSTR expression (50).

### 4.3 Molecular profile

Molecular studies in adult GEPNENs have identified recurrent genetic alterations and pathway dysregulation. Pancreatic NETs frequently harbor mutations in *MEN1*, *DAXX*, *ATRX*, and genes of the mTOR pathway (e.g., *TSC2*, *PTEN*, *PIK3CA*). These molecular findings correlate with chromosomal instability and influence prognosis (51).

Small intestinal NETs typically lack recurrent oncogenic mutations (recurrent *CDKN1B* mutations present in 8-10%) but often show chromosomal copy-number changes, particularly loss of heterozygosity at chromosome 18 (52).

NECs exhibit a distinct genomic profile with frequent—but variably prevalent—*TP53* and *RB1* inactivation depending on cell type and primary site; overall rates are lower than those typically reported in small-cell lung carcinoma (53).

In children, the molecular landscape remains far less well characterized due to small cohort sizes and limited genomic studies. Key observations include: High prevalence of hereditary predisposition syndromes, particularly MEN1, VHL, NF1, and TSC, with germline variants often preceding tumor development (13, 14, 24). This stands in contrast to adults, where hereditary syndromes account for <10% of cases. Somatic mutation profiling of pediatric NENs remains sparse, though limited reports suggest overlap with adult patterns in pancreatic NETs (e.g., MEN1 alterations), but less consistent evidence for *DAXX*/*ATRX* involvement. Pediatric NECs are so rare that robust molecular profiling data are lacking; it remains uncertain whether their genomic landscape parallels adult NEC with *TP53*/*RB1* alterations. A summary of the histological and molecular characteristics in adult and pediatric GEPNEN is presented in **Table 2**.

**Table 2:** Histologic and molecular spectrum.

Feature	Adults	Children/adolescents
Histologic categories	NET G1–G3, NEC G3	Predominantly NET G1–G2; rare NET G3, very rare NEC G3
Morphology	Broad spectrum including aggressive NECs	Mostly well-differentiated NETs with indolent course
Molecular alterations (pancreatic NETs)	*MEN1*, *DAXX*, *ATRX*, mTOR pathway mutations (*PTEN*, *TSC2*, *PIK3CA*) in pancreatic NET	Germline *MEN1*, *VHL*, *NF1*, *TSC* common in pancreatic NET; limited somatic data, overlap with adults suspected
Molecular alterations (small-intestinal NETs)	LOH chr18, few recurrent mutations (CDKN1B)	Virtually no pediatric data
NEC molecular profile	*TP53*, *RB1* inactivation to variable extent	Unknown; pediatric NEC extremely rare

NET, neuroendocrine tumor; NEC, neuroendocrine carcinoma.

Emerging data suggest that epigenetic mechanisms and tumor microenvironment differences may contribute to distinct pediatric biology, though this remains speculative. Furthermore, comprehensive sequencing studies in children are lacking, underlining the need for systematic molecular profiling within prospective registries and international collaborative trials.

## 5 Treatment strategies

### 5.1 Surgical management

General principles: In both adults and children, complete macroscopic resection (R0) is the primary curative modality for well-differentiated GEPNENs. Adult guidelines emphasize early, anatomy- and grade-adapted resection with lymph-node assessment where indicated, followed by risk-adapted surveillance. ENETS 2023–2024 organ-site updates and ASCO 2023 systemic-therapy guidance frame surgery as the cornerstone for localized disease and as part of multimodal management in advanced cases.

Registry data confirm the prognostic importance of resection in children. In the German MET pancreatic NEN cohort (1997–2024), R0/R1 resection was significantly associated with improved event-free outcomes; metastatic status at diagnosis and surgical completeness were dominant determinants of prognosis (13). For pediatric GI-NENs (non-appendiceal), overall survival is high when tumors are resectable; relapse risk clusters in those with advanced stage and incomplete resection (15).

#### 5.1.1 Pancreatic NENs

Adult ENETS guidance recommends parenchyma-sparing strategies (e.g., enucleation) for small, superficial insulinomas away from the main pancreatic duct; formal pancreatectomy (distal pancreatectomy or pancreatoduodenectomy) is used for larger or duct-adjacent lesions, or when oncologic lymphadenectomy is indicated (e.g., higher-grade, non-functional tumors). In children, surgical practice mirrors these principles with stronger preference for organ preservation to limit endocrine/exocrine insufficiency and late effects. Enucleation is typical for solitary insulinomas; limited pancreatectomy is used for non-functional or multifocal disease, with consideration of MEN1-associated multiplicity and lifelong risk of metachronous lesions.

#### 5.1.2 Gastroduodenal and colorectal NENs (excluding appendix)

Adult ENETS guidance supports limited local/segmental resection with appropriate nodal assessment for duodenal and gastric type-1/2 lesions, escalating to wider resections for type-3 gastric NETs or higher-grade tumors; colorectal NENs generally require segmental colectomy with lymph-node dissection for lesions beyond endoscopic criteria. In pediatrics, cases are few, but registry analyses show a similar pattern: most tumors are well-differentiated and resectable; endoscopic removal may be feasible for select small gastric or rectal lesions, whereas segmental surgery is preferred for larger, invasive, or higher-grade tumors.

#### 5.1.3 Small-intestine NENs

Adult recommendations favor segmental resection with systematic mesenteric nodal dissection and careful assessment of mesenteric fibrosis. True pediatric cases are rare; when encountered, the adult surgical template is generally applied in expert centers.

#### 5.1.4 Liver lesions and NEN-CUP

Liver lesions typically represent metastases. When feasible, metastasectomy/cytoreduction may be considered in liver-dominant disease after multidisciplinary review. Pediatric NEN-CUP usually presents with disseminated disease; surgery is limited to biopsy or symptom-directed procedures.

#### 5.1.5 Take-home comparison

Surgical indications are broadly concordant across ages; pediatric priorities place greater emphasis on parenchyma preservation, genetic context (MEN1, VHL, NF1, TSC), and minimizing late effects, while adult guidelines offer more granular, site-specific algorithms that are often adopted in pediatric practice for rare non-appendiceal primaries (**Table 3**).

**Table 3:** Treatment strategies: adults vs. children.

Domain	Adults (Guidelines)	Children (Evidence & adaptations)
Surgery	Standard for localized disease; site-specific algorithms	Also mainstay; stronger emphasis on organ preservation, growth/development, hereditary syndromes
Somatostatin analogues (SSA)	First-line systemic therapy in SSTR+ NETs; proven anti-proliferative effect (PROMID*, CLARINET*)	Used for symptom control and disease stabilization; well tolerated; extrapolated efficacy
Peptide receptor radionuclide therapy (PRRT)	Established after SSA failure (NETTER-1 trial*)	Increasing use in SSTR+ progressive disease; feasible in children but long-term toxicity data limited
Targeted therapy (everolimus, sunitinib)	PFS benefit in pancreatic NETs (RADIANT-3/-4, sunitinib trial*); recommended after SSA/PRRT	Off-label; used in progressive NETs in expert centers; limited pediatric data
Chemotherapy	Platinum-etoposide for NEC; CAPTEM for pancreatic NET	Reserved for NEC G3 or progressive NET G3; evidence limited to small series
Liver-directed therapies	TAE, TACE, SIRT for unresectable liver-dominant disease	Rarely used; anecdotal reports; only in expert centers
Radiotherapy	Palliative use in metastatic disease, esp. bone	Very rarely used; considered only for NEC or symptomatic metastases

NET, neuroendocrine tumor; NEC, neuroendocrine carcinoma; SSTR, somatostatin receptor; TAE, transarterial embolization; TACE, chemoembolization; SIRT, radioembolization; CAPTEM, capecitabine and temozolomide; *trial names.

### 5.2 Systemic therapies

Systemic therapy is indicated for unresectable, metastatic, or progressive disease and for persistent symptoms in functioning tumors. Adult evidence (randomized/phase-III trials and consensus guidelines) defines the therapeutic backbone; pediatric use is largely extrapolative, with growing—but still limited—cohort-level experience.

A recent review has comprehensively summarized the cumulative experience with systemic therapies in pediatric GEPNENs, encompassing SSA, PRRT, targeted agents, and chemotherapy (21).

#### 5.2.1 Somatostatin analogues

Adults: Long-acting octreotide (PROMID study) (54) significantly prolonged time-to-tumor-progression in metastatic midgut NETs. Lanreotide (CLARINET study) (55) significantly improved progression-free survival (PFS) in non-functioning grade 1–2 enteropancreatic NETs. Contemporary guidelines position SSAs as first-line therapy for SSTR-positive, well-differentiated NETs (symptom control and anti-proliferative benefit).

Pediatrics: Registry-based and review data show SSAs are well tolerated, useful for biochemical/symptom control (e.g., gastrinoma) and for disease stabilization in SSTR-positive NET G1–G2; prospective pediatric efficacy data are limited, and optimal sequencing is inferred from adult practice. Potential side effects such as pancreatic insufficiency and Vitamin B12 and D depletion warrant special consideration.

#### 5.2.2 Peptide receptor radionuclide therapy

Adults: ^177Lu-DOTATATE significantly prolongs PFS in SSTR-positive NETs, as first shown in the NETTER-1 trial for midgut primaries (56). Recent studies have expanded this evidence: NETTER-2 demonstrated benefit in previously untreated G2–3 GEP-NETs, while the COMPETE trial reported superior outcomes with ^177Lu-Edotreotide compared with everolimus (57). These data confirm PRRT as an effective option not only after SSA failure but also earlier in the treatment sequence (50).

Pediatrics: Retrospective series suggest feasibility and symptomatic benefit, with toxicity profiles comparable to adults. The prospective NETTER-P trial recently confirmed safety and preliminary efficacy of ^177Lu-DOTATATE in pediatric patients, supporting its integration into multidisciplinary care of progressive SSTR-positive disease (58).

#### 5.2.3 Targeted agents

Adults: Everolimus improved PFS in progressive pancreatic NETs (RADIANT-3) and in non-functional GI/lung NETs (RADIANT-4) (59–61). Sunitinib doubled PFS vs placebo in progressive well-differentiated pancreatic NETs. Guidelines incorporate these agents after SSA ± PRRT based on site, grade, and slope of progression.

Pediatrics: Off-label everolimus and sunitinib are used selectively for progressive NETs; published pediatric data remain limited to small series, with variable responses and manageable toxicity profiles under specialist monitoring. Choice is guided by SSTR IHC and imaging, grade/Ki-67, prior SSA/PRRT, comorbidity, and center experience.

#### 5.2.4 Cytotoxic chemotherapy

Adults: For well-differentiated NETs, temozolomide-based regimens (e.g., CAPTEM) or streptozotocin/5-FU are used especially in pancreatic NETs; for G3 NET/NEC, platinum-etoposide or alternative platinum doublets are standard. Guidelines emphasize grade-adapted selection.

Pediatrics: Chemotherapy is reserved for high-grade tumors (NET G3/NEC G3) or rapidly progressive disease refractory to SSA/PRRT/targeted agents. Pediatric evidence is sparse; platinum-etoposide is most commonly used, while temozolomide-based or streptozotocin-based schedules may be considered for selected NETs.

#### 5.2.5 Immunotherapy and other emerging approaches

Immune checkpoint inhibitors have shown limited activity in well-differentiated NETs; their use outside of trials is not recommended in either population. Adult pipelines include radiolabeled SSTR antagonists and alpha-emitters; as well as bispecific antibodies targeting DLL3 in NEC. Pediatric use remains investigational with novel RLT (21).

#### 5.2.6 Comparative summary

Adults benefit from robust RCT-level evidence (PROMID, CLARINET, NETTER-1/2, COMPETE, RADIANT-3/-4; sunitinib phase III), which structures first- and later-line sequencing. Pediatric systemic therapy is extrapolated from these data and guided by SSTR imaging, grade, clinical symptoms, and tumor growth rate, with growing—yet still limited—cohort outcomes.

### 5.3 Liver-directed therapies

Adults: In liver-dominant, unresectable, well-differentiated NET metastases, transarterial embolization (TAE), chemoembolization (TACE), and radioembolization (SIRT) are established options for cytoreduction and symptom control; no intra-arterial technique has proven superior in head-to-head RCTs, and selection is center- and patient-specific (62–64).

Pediatrics: Evidence is limited to isolated reports and very small series; decisions are highly individualized and should be confined to expert centers with pediatric interventional radiology and robust peri-procedural endocrine/nutritional support. Given growth considerations and late-effects risks, careful selection and multidisciplinary review are essential.

### 5.4 Other therapies (external-beam radiotherapy, focal ablation)

External-beam radiotherapy has a limited role for well-differentiated NETs in both adults and children, reserved for palliation of symptomatic bone/CNS metastases or select local control scenarios; it is more commonly considered for NEC. Thermal ablation (e.g., RFA/MWA) may be used as adjunctive therapy for small liver metastases in adults; pediatric experience is anecdotal.

### 5.5 Practical sequencing (comparative perspective)

Adults (guideline-based): For small-bowel NET with SSTR-positive, well-differentiated disease, the established sequence is somatostatin analogue followed by PRRT and everolimus (7). For pancreatic NET, sequencing typically follows SSA, PRRT or targeted therapy (everolimus or sunitinib), and chemotherapy; in high-volume or rapidly progressive disease—even at lower grade—cytotoxic chemotherapy may be used upfront (objective response ~40%), followed by targeted agents or PRRT (5, 6). Sequencing is tailored to grade, primary site, tumor burden, and symptoms, and decisions are individualized within multidisciplinary tumor boards, taking genetic background into account where applicable.

Pediatrics (evidence-adapted): For unresectable or metastatic disease, pediatric evidence is limited; thus, systemic management is generally adapted from adult protocols. In SSTR-positive, well-differentiated NET G1–G2, somatostatin analogues may be used; PRRT may be considered at experienced centers for progression or symptom control; targeted therapies may be options in progressive disease; chemotherapy is reserved for NET G3/NEC or rapidly progressive tumors. Given the paucity of pediatric data, no definitive sequencing can be recommended. All decisions should be individualized within pediatric tumor boards, with explicit attention to hereditary context and long-term toxicity (21).

## 6 Prognosis and long-term outcomes

### 6.1 Survival and mortality

Adults: Survival outcomes for adult GEPNENs are strongly influenced by primary site, stage, and grade. SEER and European registry analyses report 5-year overall survival (OS) of 60–90% for localized NETs, 40–70% for regionally advanced disease, and less than 30% for metastatic disease (65, 66). More recent U.S. data show ~90% 5-year OS for localized, ~85% for regional, and ~57% for distant stage, with marked site heterogeneity at distant stage—higher in small-intestinal NETs (~70%), intermediate for pancreatic NETs (~50%). and lower colorectal primaries (~30-35%) (47). For adult NEC, median OS rarely exceeds 12–18 months despite systemic therapy.

Children: Registry studies consistently demonstrate excellent survival in resected, localized pediatric GEPNENs, with 5-year OS rates exceeding 90% in pancreatic and GI NETs (15, 17, 18). Outcomes in metastatic disease are substantially worse: 40-60% in children with metastatic pancreatic NENs (13, 18, 67) Pediatric NECs remain anecdotal but, when present, have survival comparable to adult NECs (14, 18). Notably, even in advanced cases, prolonged survival has occasionally been reported after multimodal therapy including surgery, PRRT, and systemic agents, highlighting potential differences in tumor biology and treatment responsiveness between age groups.

### 6.2 Recurrence and disease course

Adults: Disease relapse after resection is common, particularly in pancreatic NETs, with recurrence rates of 30–60% depending on tumor size, grade, nodal status, and resection margins. Adult NETs often follow an indolent but chronic course, with long periods of stable disease interspersed with progression. In contrast, NECs are characterized by rapid progression and early recurrence.

Children: In pediatrics, recurrence rates appear lower in completely resected NET G1–G2, though robust data are scarce due to small numbers and limited follow-up. When recurrence occurs, it is usually distant rather than local, most often in the liver or lymph nodes. The overall disease course in children appears more indolent than in adults, even for higher Ki-67 indices, but late relapses have been reported, supporting the need for long-term surveillance. For pediatric NEN-CUP, relapse and progression are almost universal, reflecting the aggressive biology of this subgroup.

### 6.3 Long-term surveillance and quality of life

Adults: Long-term surveillance in adults is guided by ENETS/ESMO, with imaging and biochemical follow-up intervals determined by grade, site, and stage. Survivorship challenges include hormone-related syndromes (functional tumors, carcinoid heart disease), treatment-related late effects (renal/hepatic from PRRT, metabolic from surgery), and psychosocial impact of chronic disease (26).

Children: In pediatric patients, lifelong follow-up is essential, even after complete resection, given the potential for late recurrence and the high prevalence of hereditary syndromes. Surveillance strategies are less standardized than in adults; expert consensus supports annual clinical review and imaging every 1–2 years for at least 10 years, with shorter intervals in higher-risk cases (large tumors, nodal or distant disease, higher grade).

Long-term survivorship issues are particularly relevant in children: Endocrine late effects from pancreatic resection (diabetes, exocrine insufficiency), hormonal sequelae in functional NETs, potential renal/gonadal toxicity from PRRT, psychosocial challenges related to chronic disease, hereditary risk, and transition to adult care.

Quality of life studies in pediatric GEPNENs are lacking; extrapolation from adult NET cohorts suggests that chronic treatment, surveillance burden, and anxiety about recurrence significantly impact well-being. The longer expected lifespan of pediatric patients amplifies the importance of minimizing late effects and integrating psycho-oncology into care.

## 7 Guideline recommendations and current clinical practice

### 7.1 Development of pediatric-specific guidelines

In adults, care pathways for GEPNENs are codified by detailed, organ-site–specific recommendations that integrate histology/grade, stage, SSTR status, and patient factors. By contrast, pediatric guidance remains limited due to extreme rarity, a paucity of prospective data, and heterogeneous case-mix across centers. As a result, management in children largely adapts adult algorithms, with pediatric modifications for: (i) growth and developmental considerations, (ii) hereditary cancer predisposition syndromes (more frequent in childhood), and (iii) late-effects minimization (organ-sparing surgery, judicious use of radiation and alkylators).

Over the past decade, European collaborative structures—EXPeRT (European Cooperative Study Group for Paediatric Rare Tumours), ERN PaedCan, and EndoERN—have expanded the portfolio of European Standard Clinical Practice (ESCP) documents for very rare tumors (68). Within NENs, a first pediatric ESCP was completed for appendiceal NEN, while focused registry analyses and critical reviews have begun to outline pediatric-adapted recommendations. Together, these efforts form the substrate for a consolidated, site-spanning pediatric GEPNEN guidance.

Implication: The field now requires (i) a unified pediatric ESCP for non-appendiceal GEPNENs and (ii) integration of pediatric-feasible metrics (age-appropriate imaging, sedation needs, endocrine follow-up, fertility preservation) into decision pathways that were originally validated in adults.

### 7.2 European consensus and harmonization efforts

Adult guideline ecosystems (ENETS, ESMO, ASCO, NANETS) offer granular, site-specific algorithms for diagnostic staging (including 68Ga-DOTA PET), histopathological work-up (grading per WHO 2019), and sequencing of surgery → SSA → PRRT/targeted agents → cytotoxic therapy, with selective use of liver-directed procedures. These frameworks are widely implemented in European NET centers (5–12).

Pediatric harmonization is advancing along three axes:

– Centralized review and MDT structures: Systematic presentation of pediatric NEN cases at multidisciplinary tumor boards that include pediatric oncology, endocrine surgery, gastroenterology, nuclear medicine, pathology, interventional radiology, and genetics. Complex cases (e.g., high-grade disease, PRRT candidates, multifocal MEN1) should be managed in or in collaboration with expert NET centers.
– Common diagnostic standards: Routine use of contrast-enhanced MRI (preferred in children), 68Ga-DOTA PET/CT or PET/MRI for SSTR-positive tumors, and FDG-PET for NET G3/NEC; expert pathology with Ki-67-based grading (NET G1–G3 vs NEC); and universal germline evaluation for MEN1/VHL/NF1/TSC (given higher pediatric prevalence).
– Pediatric-adapted therapeutic pathways: Adoption of adult-validated modalities with child-specific safeguards: organ-sparing pancreatic surgery when oncologically safe; SSA first-line in SSTR-positive, well-differentiated disease; PRRT in expert settings with long-term renal/gonadal surveillance; everolimus/sunitinib in selected progressive cases; and platinum-based chemotherapy reserved for NEC or rapidly progressive NET G3. Liver-directed procedures are considered case-by-case in specialized centers.

### 7.3 Risk stratification: pediatric vs. adult frameworks

Adult algorithms stratify risk using WHO grade (Ki-67, mitotic index), TNM stage, tumor site, SSTR expression, and dynamic factors such as tumor growth rate and symptom burden. These variables remain relevant in children, but pediatric care benefits from additional, age-specific layers:

– Germline predisposition (MEN1, VHL, NF1, TSC): informs multiplicity risk (e.g., MEN1 pancreatic microadenomatosis), dictates life-long surveillance, and influences thresholds for intervention (e.g., parenchyma-sparing resections, selective node assessment).
– Growth and development: prioritization of strategies that minimize endocrine insufficiency (enucleation where feasible), reduce cumulative radiation (MRI preference; careful PRRT eligibility and dosing), and preserve fertility (pre-treatment counselling).
– Long-term toxicity horizon: conservative use of alkylators and intra-arterial therapies; proactive renal protection around PRRT; structured survivorship plans.

A pragmatic pediatric risk framework therefore layers WHO grade + SSTR status + stage on top of germline status and anticipated late-effects risk, to guide sequencing (e.g., SSA before PRRT/targeted agents in indolent SSTR-positive NET G1–G2; early escalation for NET G3; chemotherapy front-line for NEC).

### 7.4 Transition of care

Because pediatric GEPNENs often require lifelong surveillance, a structured transition to adult NET services is essential. Best practice includes:

– Early planning (beginning at ~16–17 years): joint pediatric–adult clinics, written care plans summarizing diagnosis, grade, stage, treatments received (including cumulative radiation/PRRT dosimetry), genetic findings, and specific late-effects risks.
– Defined surveillance schedule post-transition: periodic clinical review, endocrine/metabolic assessment (especially after pancreatic surgery or functional tumors), and risk-adapted imaging intervals (usually 6–24 months depending on grade, stage, and tempo).
– Psychosocial and fertility support: address hereditary implications for relatives, family planning, and access to adult genetic counselling; integrate psycho-oncology to mitigate anxiety and treatment burden in chronic disease.

### 7.5 Practical summary (comparative perspective)

Alignment: Core pillars—WHO grading, TNM staging, SSTR-guided imaging, early surgery for localized disease, SSA as first-line systemic therapy, PRRT/targeted agents for progression, and chemotherapy for high-grade tumors—are shared across adult and pediatric practice.

Pediatric adaptations: Higher prevalence of hereditary syndromes, organ-sparing imperatives, and long survivorship horizons necessitate tailored thresholds for intervention and long-term toxicity mitigation. Centralization and MDT review are especially critical given very low case volumes.

Harmonization agenda: Europe is well placed to formalize a pan-pediatric GEPNEN ESCP (non-appendiceal) that embeds adult evidence where appropriate, codifies pediatric-specific safeguards, and links seamlessly to the existing appendiceal NET ESCP.

## 8 Conclusion

Pediatric GEPNENs are exceptionally rare, biologically distinct from adult disease, and strongly enriched for hereditary cancer predisposition syndromes. While localized, well-differentiated NETs often have an excellent prognosis after resection, outcomes for metastatic and high-grade tumors remain poor.

Current management in children relies heavily on adaptation of adult guidelines, with necessary modifications to minimize long-term sequelae and to account for genetic context and developmental considerations. This comparative review highlights the urgent need for pediatric-specific recommendations, harmonized European practice, and international collaborative studies to address persisting evidence gaps and to improve long-term outcomes.
